# Myricetin Protects Cells against Oxidative Stress-Induced Apoptosis via Regulation of PI3K/Akt and MAPK Signaling Pathways

**DOI:** 10.3390/ijms11114348

**Published:** 2010-11-02

**Authors:** Kyoung Ah Kang, Zhi Hong Wang, Rui Zhang, Mei Jing Piao, Ki Cheon Kim, Sam Sik Kang, Young Woo Kim, Jongsung Lee, Deokhoon Park, Jin Won Hyun

**Affiliations:** 1 School of Medicine and Applied Radiological Science Research Institute, Jeju National University, Jeju 690-756, Korea; E-Mails: legna48@hanmail.net (K.A.K); wzh407@hotmail.com (Z.H.W); zhangrui26@hotmail.com (R.Z); mjpiao@hanmail.net (M.J.P); svv771@hanmail.net (K.C.K); 2 College of Pharmacy, Seoul National University, Seoul 110-460, Korea; E-Mail: sskang@snu.ac.kr (S.S.K); 3 Biospectrum Life Science Institute, Gunpo 435-833, Korea; E-Mails: ywkim@biospectrum.com (Y.W.K); jslee@biospectrum.com (J.L); pdh@biospectrum.com (D.P)

**Keywords:** myricetin, cytoprotective effect, oxidative stress

## Abstract

Recently, we demonstrated that myricetin exhibits cytoprotective effects against H_2_O_2_-induced cell damage via its antioxidant properties. In the present study, myricetin was found to inhibit H_2_O_2_-induced apoptosis in Chinese hamster lung fibroblast (V79-4) cells, as shown by decreased apoptotic bodies, nuclear fragmentation, sub-G_1_ cell population, and disruption of mitochondrial membrane potential (Δψ*_m_*), which are increased in H_2_O_2_-treated cells. Western blot data showed that in H_2_O_2_-treated cells, myricetin increased the level of Bcl-2, which is an anti-apoptotic factor, and decreased the levels of Bax, active caspase-9 and -3, which are pro-apoptotic factors. And myricetin inhibited release of cytochrome c from mitochondria to cytosol in H_2_O_2_-treated cells. Myricetin-induced survival correlated with Akt activity, and the rescue of cells by myricetin treatment against H_2_O_2_-induced apoptosis was inhibited by the specific PI3K (phosphoinositol-3-kinase) inhibitor. Myricetin-mediated survival also inhibited the activation of p38 mitogen activated protein kinase (MAPK) and c-Jun *N*-terminal kinase (JNK), which are members of MAPK. Our studies suggest that myricetin prevents oxidative stress-induced apoptosis via regulation of PI3K/Akt and MAPK signaling pathways.

## 1. Introduction

Reactive oxygen species (ROS) are ions or very small molecules that include oxygen ions, and are produced as normal products of cellular metabolism [1]. However, elevated production of ROS increases oxidative stress, leading to cellular dysfunction and cell death [2]. ROS play an important role in apoptosis induction under both physiologic and pathologic conditions [3,4]. The phosphoinositol-3-kinase (PI3K)/Akt signaling pathway is considered to be one of the survival pathways within cells [5]. It is activated by many types of cellular stimulation and regulates fundamental cellular functions such as cell growth, proliferation, and cell cycle [6,7]. It has been shown to play a major role in the prevention of apoptosis induced by oxidative stress [8,9]. In addition, ROS that cause oxidative stress are known to activate members of the mitogen activated protein kinase (MAPK) family [10]. MAPKs are important mediators of signal transduction, and play a key role in the regulation of cell growth, proliferation, differentiation, and apoptosis. The MAPK comprise three subfamilies: extracellular signal-regulated kinase (ERK), c-jun *N*-terminal kinase (JNK), and p38 MAPK [11]. In general, ERK induces a survival or proliferation signal, while JNK and p38 MAPK induce an apoptosis signal under stressful conditions [12,13]. Activated ERK, JNK, and p38 MAPK modulate the phosphorylation of transcription factors, ultimately leading to changes in gene expression profiles that encode for defense against cellular oxidative stress [14,15].

Myricetin (3,3’,4’5,5’,7-hexahydroxylflavone) is a natural flavonoid, found in many fruits, vegetables, and herbs. We have recently shown that myricetin could act as a direct antioxidant that scavenges or quenches oxygen free radicals, and as an indirect antioxidant that induces antioxidant enzymes to protect cells against H_2_O_2_-induced cell damage [16].

In this study, we investigated the anti-apoptotic effect of myricetin against oxidative stress and the involvement of the PI3K/Akt and MAPK pathways.

## 2. Results and Discussion

### 2.1. Myricetin Inhibits H_2_O_2_-Induced Cell Death

**Figure 1.**
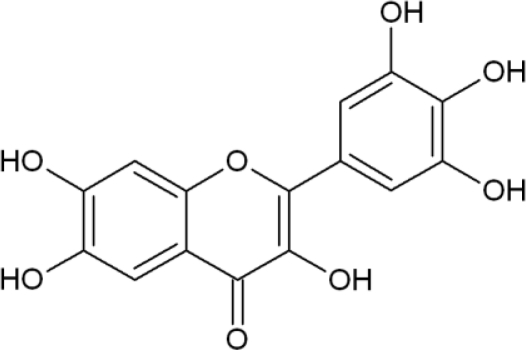
Chemical structure of myricetin (3,3’,4’5,5’,7-hexahydroxylflavone).

**Figure 2.**
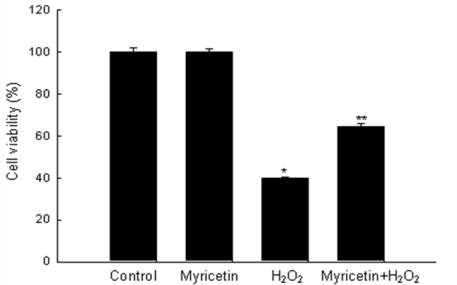
Effect of myricetin on H_2_O_2_-induced cell death. Cells were treated with myricetin at 30 μM. After 1 h, 1 mM of H_2_O_2_ was added to the plate, and cell viability was determined after an incubation of 24 h by the MTT assay. *Significantly different from control cells (*p* < 0.05). **Significantly different from H_2_O_2_-treated cells (*p* < 0.05).

Oxidative stress is a major cause of cellular injuries in a variety of human disorders [17,18]. Considerable efforts have been made to find natural antioxidants with protective potential against oxidative stress. Myricetin (Figure 1) is a potent flavonoid antioxidant and we have recently shown that myricetin can protect cells against H_2_O_2_-induced cell damage [16]. H_2_O_2_ has been extensively used as an inducer of oxidative stress, resulting in cell death including apoptosis [19–21]. The effect of myricetin on cell survival in H_2_O_2_-treated cells was measured at 24 h using the MTT test. Myricetin at 30 μM did not show cytotoxicity compared to control (Figure 2). Treatment with myricetin increased cell survival to 65% compared to 40% survival with H_2_O_2_ treatment. We did experiment to elucidate the up-take of myricetin into cells; cells were pre-treated with myricetin for 24 hours, removed the medium, then washed, and added new media without myricetin. After addition of H_2_O_2_ treatment, the cell viability was detected using MTT test after 24 hours. The cell viability results were consistent with results of continued incubation of myricetin in Figure 2 (and data not shown). These results suggest that myricetin was up-taken into cells and showed a cytoprotective effect on H_2_O_2_-induced cell damage.

### 2.2. Protective Effect of Myricetin against Apoptosis Induced by H_2_O_2_

**Figure 3.**
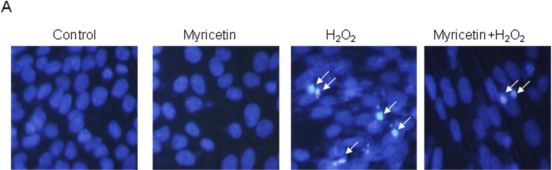

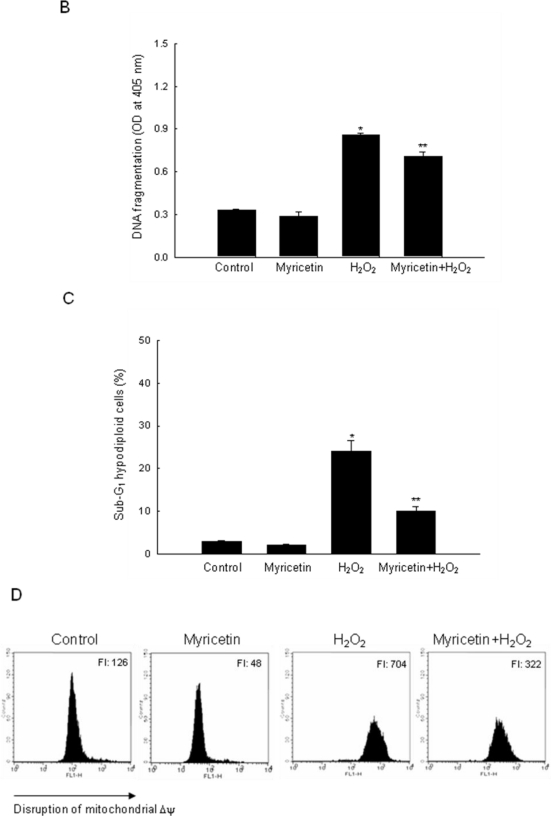

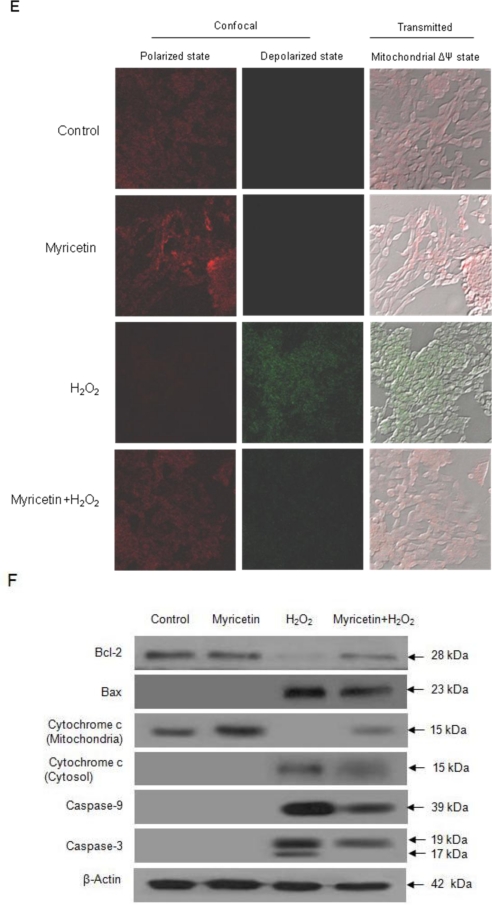
Effect of myricetin on H_2_O_2_-induced apoptosis (**A**) Apoptotic body formation was observed under a fluorescent microscope after Hoechst 33342 staining. The apoptotic bodies are indicated with arrows. (**B**) DNA fragmentation was quantified by ELISA. *Significantly different from control cells (*p* < 0.05). **Significantly different from H_2_O_2_-treated cells (*p* < 0.05). (**C**) The apoptotic sub-G_1_ DNA content was detected by flow cytometry after propidium iodide staining. *Significantly different from control cells (*p* < 0.05). **Significantly different from H_2_O_2_-treated cells (*p* < 0.05). The mitochondrial Δψ*_m_* was assessed after JC-1 staining by (**D**) flow cytometer, and (**E**) confocal microscope. (**F**) The cell lysates were electrophoresed, and detected Bcl-2, Bax, cytochrome c, caspase-3, and -9 by specific antibody.

In the present study, we found that myricetin decreased cell death induced by H_2_O_2_, and this result was further confirmed by apoptotic observation. To evaluate the cytoprotective effect of myricetin on apoptosis induced by H_2_O_2_, the nuclei of cells were stained with Hoechst 33342 and assessed by microscopy. The microscopic pictures in Figure 3A show that the control cells had intact nuclei, while the H_2_O_2_-treated cells exhibited significant nuclear fragmentation, which is indicative of apoptosis. However, when the cells were treated with myricetin for one hour prior to H_2_O_2_ treatment, reduced nuclear fragmentation was observed. These data suggest that H_2_O_2_-treated cells displayed typical features of apoptosis with fragmented nuclei; however, myricetin inhibited these morphological changes. In addition, H_2_O_2_-treated cells had increased levels of cytoplasmic histone-associated DNA fragmentation compared to the control group. However, myricetin decreased the degree of DNA fragmentation (Figure 3B). The protective effect of myricetin against apoptosis was also confirmed by an apoptotic sub-G_1_ DNA analysis. As shown in Figure 3C, an analysis of the DNA content in H_2_O_2_-treated cells revealed a 24% increase in the apoptotic sub-G_1_ DNA content. Moreover, myricetin decreased the apoptotic sub-G_1_ DNA content to 10%. Mitochondrial membrane potential (Δψ*_m_*) analysis showed that the level of Δψ*_m_* loss was increased in H_2_O_2_-treated cells, as substantiated by an increase in fluorescence with the JC-1 dye, however, myricetin recovered the level of Δψ*_m_* loss (Figure 3D). The flow cytometric data was consistent with the image analysis data; the red fluorescence of JC-1 (JC-1 aggregated form, indicative of mitochondrial polarization) was decreased in H_2_O_2_-treated cells, whereas the green fluorescence (JC-1 monomer form, indicative of mitochondrial depolarization) was greatly increased. Myricetin blocked the loss of Δψ*_m_* after H_2_O_2_ treatment, as shown in Figure 3E. These results suggest that myricetin protects cells via inhibition of the mitochondria dependent apoptosis pathway. Mitochondria act as an important apparatus for signals during apoptosis, and the loss of mitochondrial integrity can be prompted or inhibited by many regulators of apoptosis [22]. In many cases, oxidative stress induces caspase activation through cytochrome c release from the mitochondrial inter-membrane space into the cytosol [23]. H_2_O_2_ treatment increased the expressions of Bax, active caspase-9, and -3, which are pro-apoptotic factors, but decreased the expression of Bcl-2, which is an anti-apoptotic factor. And H_2_O_2_ treatment also increased the release of cytochrome c from mitochondria to cytosol (Figure 3F). Myricetin inhibited the H_2_O_2_-induced release of mitochondrial cytochrome c. During the apoptotic process, Bcl-2 prevents the opening of the mitochondrial membrane pores, whereas Bax induces the opening of membrane pores [24]. Therefore, the blocked loss of Δψ*_m_* by myricetin may be the result of Bcl-2 up-regulation, and Bax down-regulation.

### 2.3. Involvement of PI3K/Akt and MAPKs in the Anti-apoptotic Effect of Myricetin from H_2_O_2_ Treatment

**Figure 4.**
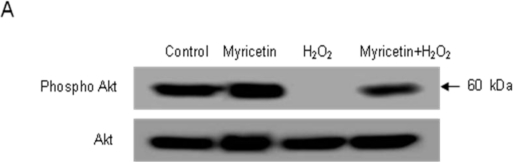

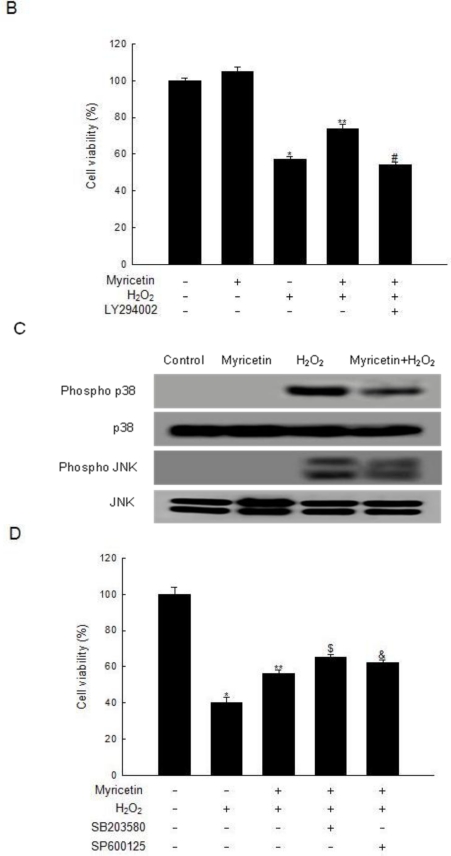
Effect of myricetin on PI3K and MAPK signaling pathways. (**A**) Protein expression of phospho Akt was determined by western blot analysis. (**B**) After treatment with LY294002, myricetin or/and H_2_O_2_, the cell viability was assessed by MTT assay. *Significantly different from control cells (*p* < 0.05). **Significantly different from H_2_O_2_-treated cells (*p* < 0.05). ^#^Significantly different from myricetin+H_2_O_2_-treated cells (*p* < 0.05). (**C**) Protein expression of phospho JNK and p38 was determined by Western blot analysis. (**D**) After treatment with SB203580 or SP600125, myricetin or/and H_2_O_2_, the cell viability was determined by the MTT assay. *Significantly different from control cells (*p* < 0.05). **Significantly different from H_2_O_2_-treated cells (*p* < 0.05). ^$^ and ^&^ significantly different from myricetin+H_2_O_2_-treated cells (*p* < 0.05).

Activation of PI3K and its downstream effector Akt has also been shown to suppress apoptosis and promote cell survival [25–27]. It has been shown that activation of PI3K leads to the phosphorylation and activation of Akt, which promotes cell survival by enhancing the expression of anti-apoptotic proteins and inhibiting the activity of pro-apoptotic proteins [28,29]. To further elucidate the mechanism of myrcetin-mediated cell survival, we examined the activation of Akt, a major signaling enzyme involved in cell survival against oxidative stress. Western blot analysis showed that myricetin increased Akt phosphorylation compared to the decreased Akt phosphorylation with H_2_O_2_ treatment (Figure 4A). In addition, LY294002 (a specific PI3K inhibitor) attenuated the protective effect of myricetin against H_2_O_2_-induced cytotoxicity (Figure 4B). These results suggest that PI3K/Akt is activated by myricetin and rescues cells from H_2_O_2_-induced apoptosis. Because p38 MAPK and JNK play important roles in modulating apoptosis, we next examined the effects of myricetin on the activation of p38 MAPK and JNK. The activation states of MAPKs were determined by measuring the expression of their phosphorylated forms. H_2_O_2_ treatment increased phosphorylation of p38 MAPK and JNK compared to that in control cells, however, myricetin decreased their phosphorylation (Figure 4C). In addition, SB203580 (a specific p38 MAPK inhibitor) and SP600125 (a specific JNK inhibitor) maintained the protective effect of myricetin against H_2_O_2_-induced cytotoxicity (Figure 4D). These results suggest that myricetin provides a cytoprotective effect against H_2_O_2_-induced apoptosis via inhibition of p38 MAPK and JNK.

## 3. Conclusions

Our studies demonstrated that myricetin showed a cytoprotective effect against oxidative stress-induced mitochondrial dependent and caspases dependent apoptosis via regulation of PI3K/Akt, p38 MAPK and JNK signaling pathways.

## 4. Experimental Section

### 4.1. Reagents

Myricetin, Hoechst 33342, propidium iodide, and [3-(4,5-dimethylthiazol-2-yl)-2, 5-diphenyltetrazolium] bromide (MTT) were purchased from Sigma Chemical Company (St. Louis, U.S.). 5,5’,6,6’-tetrachloro-1,1’,3,3’-tetraethyl-benzimidazolylcarbocyanine iodide (JC-1) was purchased from Invitrogen Corporation (Carlsbad, U.S.). The primary anti-Bcl-2, -Bax, -cytochrome c, -caspase-9, -caspase-3, -Akt, -phospho Akt, -ERK, -phospho ERK, -JNK, -phospho JNK, -p38, and -phospho p38 antibodies were purchased from Cell Signaling Technology (Beverly, U.S.). LY294002, SB203580, and SP600125 were purchased from Calbiochem (San Diego, U.S.).

### 4.2. Cell Culture

Chinese hamster lung fibroblasts (V79-4) cells from the American Type Culture Collection (Rockville, U.S.) were maintained at 37 °C in an incubator with a humidified atmosphere of 5% CO_2_ and cultured in Dulbecco’s Modified Eagle’s medium containing 10% heat-inactivated fetal calf serum, streptomycin (100 μg/mL) and penicillin (100 units/mL).

### 4.3. Cell Viability

The effect of myricetin on the cell viability was determined by the MTT assay [30]. The cells were seeded in a 96 well plate at a concentration of 1 × 10^5^ cells/mL, and 16 h after plating, were treated with 30 μM of myricetin, and 1 h later, 1 mM H_2_O_2_ was added to the plate and incubated for an additional 24 h at 37 °C. Fifty microliters of MTT stock solution (2 mg/mL) was then added to each well in a total reaction volume of 200 μL. After incubating for 4 h, the plate was centrifuged at 800 × g for 5 min and the supernatants were aspirated. The formazan crystals in each well were dissolved in 150 μL DMSO and the A_540_ was read on a scanning multi-well spectrophotometer. To determine the effects of LY294002, SB203580, and SP600125 on cell viability, cells were pretreated with inhibitors for 1 h, followed by 1 h of incubation with myricetin and exposure to 1 mM H_2_O_2_ for 24 h, and cell viability was then measured using the MTT test.

### 4.4. Nuclear Staining with Hoechst 33342

The cells were treated with 30 μM of myricetin. After 1 h, 1 mM of H_2_O_2_ was added to the plate, and the mixture was incubated for 24 h. 1.5 μL of Hoechst 33342 (stock 10 mg/mL), a DNA specific fluorescent dye, was added to each well and incubated for 10 min at 37 °C. The stained cells were then observed under a fluorescent microscope, which was equipped with a CoolSNAP-Pro color digital camera to examine the degree of nuclear condensation.

### 4.5. DNA Fragmentation

Cellular DNA fragmentation was assessed by using a cytoplasmic histone-associated DNA fragmentation kit from Roche Diagnostics (Mannheim, Germany) according to the manufacturer’s instructions.

### 4.6. Flow Cytometry Analysis

Flow cytometry was performed to determine the content of apoptotic sub G_1_ hypo-diploid cells [31]. The cells were harvested, and fixed in 1 mL of 70% ethanol for 30 min at 4 °C. The cells were washed twice with phosphate buffered saline (PBS), and then incubated for 30 min in the dark at 37 °C in 1 mL of PBS containing 100 μg propidium iodide and 100 μg RNase A. Flow cytometric analysis was performed and the proportion of sub G_1_ hypo-diploid cells was assessed by the histograms generated using the computer program, Cell Quest and Mod-Fit.

### 4.7. Mitochondrial Membrane Potential (ΔΨ) Analysis

The cells were harvested, washed, and suspended in PBS containing JC-1 (10 μg/mL). After 15 min of incubation at 37 °C, the cells were washed, suspended in PBS and analyzed by flow cytometery [32]. For image analysis, the cells were loaded with JC-1, and incubated for 30 min at 37 °C. Cells were washed and the stained cells were mounted onto a microscope slide with mounting medium (DAKO, Carpinteria, U.S.). Microscopic images were collected using the Laser Scanning Microscope 5 PASCAL program (Carl Zeiss, Jena, Germany) on a confocal microscope.

### 4.8. Western Blot

The cells were harvested, washed twice with PBS, lysed on ice for 30 min in 100 μL of lysis buffer [120 mM NaCl, 40 mM Tris (pH 8), 0.1% NP 40] and then centrifuged at 13,000 × g for 15 min. The supernatants were collected from the lysates and the protein concentrations were determined. Aliquots of the lysates (40 μg of protein) were boiled for 5 min and electrophoresed in 10% sodium dodecylsulfate-polyacrylamide gel. The proteins in the gels were transferred onto nitrocellulose membranes (Bio-Rad, Hercules, U.S.), which were then incubated with the primary antibodies. The membranes were subsequently incubated with the secondary immunoglobulin-G-horseradish peroxidase conjugates (Pierce, Rockford, U.S.). Protein bands were detected using an enhanced chemiluminescence Western blotting detection kit (Amersham, Little Chalfont, Buckinghamshire, U.K.), and then exposed to X-ray film.

### 4.9. Statistical Analysis

All values were expressed as means ± standard error of the mean (SEM). The results were subjected to analysis of variance (ANOVA) using Tukey’s test to analyze differences. *p* < 0.05 were considered significant.
