# Correction: Kang, K.A.; *et al*., Myricetin Protects Cells against Oxidative Stress-Induced Apoptosis via Regulation of PI3K/Akt and MAPK Signaling Pathways. *Int. J. Mol. Sci.* 2010, *11*, 4348–4360

**DOI:** 10.3390/ijms16011482

**Published:** 2015-01-08

**Authors:** Kyoung Ah Kang, Zhi Hong Wang, Rui Zhang, Mei Jing Piao, Ki Cheon Kim, Sam Sik Kang, Young Woo Kim, Jongsung Lee, Deokhoon Park, Jin Won Hyun

**Affiliations:** 1 School of Medicine and Applied Radiological Science Research Institute, Jeju National University, Jeju 690-756, Korea; E-Mails: legna48@hanmail.net (K.A.K.); wzh407@hotmail.com (Z.H.W.); zhangrui26@hotmail.com (R.Z.); mjpiao@hanmail.net (M.J.P.); svv771@hanmail.net (K.C.K.); 2 College of Pharmacy, Seoul National University, Seoul 110-460, Korea; E-Mail: sskang@snu.ac.kr; 3 Biospectrum Life Science Institute, Gunpo 435-833, Korea; E-Mails: ywkim@biospectrum.com (Y.W.K.); jslee@biospectrum.com (J.L.); pdh@biospectrum.com (D.P.)

The authors want to change Figure 1 of the paper published in *IJMS* [1]. In Figure 1, 5-position of OH was at 6-position. Therefore, Figure 1 is revised as follows. The authors would like to apologize for any inconvenience caused to the readers by this change.

**Figure 1:**
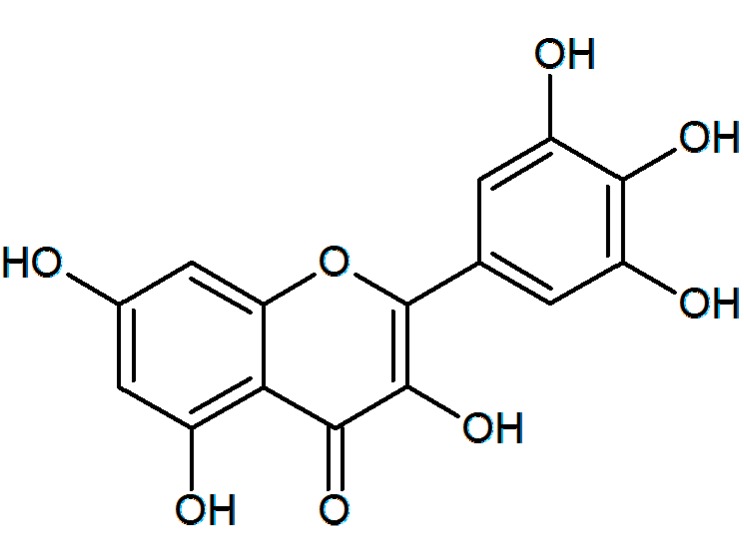
Chemical structure of myricetin (3,3',4',5,5',7-hexahydroxyflavone).
